# Pregnancy and glycemic outcomes with hybrid closed‐loop insulin delivery versus sensor‐augmented pump therapy among women with type 1 diabetes

**DOI:** 10.1002/pmf2.70392

**Published:** 2026-08-31

**Authors:** Annabeth Brewton, Radhika Pandit, Jiqiang Wu, Natasha Santhanam, Ellena Privitera, Shaylyn Vickers, Steven Gabbe, William A. Grobman, Mark B. Landon, Christine P. Field, Elizabeth O. Buschur, Kartik K. Venkatesh

**Affiliations:** ^1^ Division of Maternal Fetal Medicine Department of Obstetrics and Gynecology The Ohio State University Columbus Ohio USA; ^2^ Department of Obstetrics and Gynecology The Ohio State University Columbus Ohio USA; ^3^ Division of Maternal Fetal Medicine Department of Obstetrics and Gynecology Brown University Providence Rhode Island USA; ^4^ Division of Endocrinology Metabolism, and Diabetes Department of Medicine The Ohio State University Columbus Ohio USA

**Keywords:** adverse pregnancy outcomes, glycemic control, hybrid closed loop therapy, insulin pump, pregnancy, type 1 diabetes

## Abstract

**Objective:**

Pregnant women with type 1 diabetes (T1D) increasingly utilize hybrid closed‐loop (HCL) pumps, but *pregnancy‐specific* algorithms in many countries including the United States are not available. We compared pregnancy and glycemic outcomes between those intending to use off‐label automated HCL versus manual sensor‐augmented pump therapy (SAPT).

**Methods:**

We conducted a single‐center retrospective study from 2022 to 2025. The exposure was intent to use HCL using non‐pregnancy algorithms versus SAPT (reference) at the start of pregnancy. Outcomes included pregnancy outcomes (cesarean delivery, preeclampsia, preterm birth, small‐ and large‐for‐gestational‐age, neonatal intensive care unit admission, neonatal hypoglycemia, neonatal hyperbilirubinemia, and respiratory distress syndrome) and glycemic measures the week before delivery (hemoglobin A1c and continuous glucose monitor metrics including time in range within a pregnancy‐specific range [63–140 mg/dL], time above and below range, standard deviation, and mean glucose). An Inverse‐Probability‐of‐Treatment Weighting analytic approach was used.

**Results:**

Among 154 women with T1D, 68.2% intended to use HCL and 31.8% SAPT when initiating prenatal care (median gestational age of 8.0 weeks [interquartile range {IQR}, 7.3, 9.3]). In adjusted analyses, women using HCL had neonates with a higher birthweight (3640 vs. 3430 g; adj. beta, 271.98; 95% CI, 0.31, 543.65) and mean sensor glucose the week prior to delivery (150.0 vs. 145.0 mg/dL; adj. beta, 13.36; 95% CI, 0.79, 25.93) and lower time below range compared with those using SAPT (2.0 vs. 4.0%; adj. beta, −3.73; 95% CI, −6.52, −0.93). Other pregnancy and glycemic outcomes did not differ between groups.

**Conclusion:**

Among a US cohort with T1D enrolled in a prenatal and diabetes care program, pregnancy and glycemic outcomes were similar between those using off‐label HCL versus SAPT. Evaluating how HCL with pregnancy‐specific algorithms can be used to promote maternal and child health could be most helpful for pregnant women with T1D and should be a research priority.

## 1 INTRODUCTION

Women with type 1 diabetes (T1D) increasingly utilize hybrid closed‐loop (HCL) insulin pump therapy with continuous glucose monitoring (CGM) [1]. Recent international trials demonstrate that pregnancy‐specific automated HCL provides significantly improved glycemic control, although these studies were not powered to identify meaningful differences in pregnancy outcomes [2, 3, 4]. However, pregnancy‐specific algorithms are currently not available in the United States [1, 5].

Women with T1D using automated HCL in the United States who become pregnant must decide whether to continue off‐label use or transition to manual sensor‐augmented pump therapy [SAPT] [6]. HCL offers an advanced, automated system for insulin delivery using real‐time CGM, while SAPT provides a more traditional, user‐controlled approach combining a pump where the user must manually adjust insulin based on CGM data. Because current HCL systems are unable to adapt to placentally mediated insulin resistance, it is possible that women may need to transition from HCL to SAPT to achieve adequate glycemic management, but the outcomes for those intending to use HCL and the frequency of this transition to SAPT remain uncertain [5]. Contemporary observational data can inform patient and provider decisions regarding potential risks and benefits of currently available HCL systems in pregnancy [6].

We compared pregnancy and glycemic outcomes between women with T1D intending to use off‐label HCL versus SAPT at the start of pregnancy.

## 2 METHODS

We conducted a retrospective analysis of pregnant women with T1D enrolled in an integrated prenatal and diabetes care program at The Ohio State University, a US tertiary care center from January 2022 to December 2025. Hemoglobin A1c [HbA1] was checked as part of standard of care generally once per trimester, and CGM data were downloaded for review about every 1–2 weeks to titrate insulin. To be eligible for analysis, women had to be currently using an HCL insulin pump (including a Tandem t:slim X2®, Omnipod 5®, or Medtronic MiniMed 780G® device), have entered prenatal care <14 weeks’ gestation, and have delivered >20 weeks’ gestation. Women with multiple pregnancies were included in this analysis.

When initiating prenatal care, women using HCL were counseled that they could use HCL “off label” and rely on non‐pregnancy algorithms or they could switch to SAPT. Those who chose HCL were recommended to use the lowest available glucose target, which varied between 100 and 110 mg/dL depending on the HCL device. Those who changed to SAPT were recommended to follow the pregnancy‐specific time in range (TIR) of 63–140 mg/dL [7].

At the initial prenatal visit and at follow‐up, patients and providers engaged in shared decision‐making. Women were counseled about current guidance about using off‐label HCL with non‐pregnancy‐specific algorithms versus transitioning to SAPT. Women using both HCL and SAPT were advised to set their pump to the lowest allowable glucose target and to set their CGM target range to pregnancy‐specific recommendations of TIR 63 to 140 mg/dL. Women who chose to use HCL were counseled on adjunctive measures (i.e., fake carbs, intentional periods of SAPT, and sleep mode), as were those who chose to use SAPT (i.e., lower glucose targets and adjusting and extending basal rates).

The primary exposure for this analysis was, at initiation of prenatal care, intent to continue to use off‐label HCL versus changing to manual SAPT. Those who had ≥70% of time in automated mode and planned to continue to use HCL were categorized as HCL [2]. We secondarily compared women who used HCL at any timepoint in pregnancy (i.e., those who intended to use HCL at initiation of prenatal care plus those who used HCL later in pregnancy) versus those who used SAPT only.

Outcomes included glycemic measures the week before delivery (i.e., hemoglobin A1c [HbA1c] most proximate to delivery and CGM metrics the week before delivery including TIR within a pregnancy‐specific range [63–140 mg/dL], time above and below range [TAR, TBR], standard deviation, and mean glucose), maternal (cesarean delivery, preeclampsia) and neonatal outcomes (preterm birth [PTB], small‐for‐gestational‐age [SGA] and large‐for‐gestational‐age [LGA], intensive care unit [NICU] admission, hypoglycemia [glucose <40 to 50 mg/dL per 24 h after birth or oral or IV glucose therapy), hyperbilirubinemia (per postnatal age‐specific nomogram or phototherapy), and respiratory distress syndrome (per clinical diagnosis). These outcomes were selected based on a core outcome set.

For the primary analysis, we compared outcome data between those who intended to use HCL versus SAPT at the start of pregnancy. An Inverse‐Probability‐of‐Treatment Weighting (IPTW) analytic approach was used to address confounding by indication using logistic regression. The propensity score model adjusted for insulin pump type, duration of T1D, and early pregnancy A1c. Covariate balance was assessed before and after IPTW by standardized mean differences for all covariates. Generalized estimating equations using modified Poisson regression that clustered for women who provided data from more than one pregnancy to account for inter‐person correlation were used to calculate unadjusted and adjusted risk ratios (RRs). Missing covariates were imputed using Multivariate Imputation by Chained Equations (MICE) with 30 imputations and included all baseline covariates in the adjusted model above. All analyses were conducted in R software (version 4.1.1; R Core Team). The Ohio State University Institutional Review Board approved the study.

## 3 RESULTS

During the study period, there were 154 deliveries to 125 pregnant women with T1D who met inclusion criteria. At the initiation of prenatal care for these 154 pregnancies (median gestational age of 8.0 weeks [interquartile range {IQR}, 7.3, 9.3]), 68.2% (*n* = 105) of women intended to use HCL and 31.8% (*n* = 49) SAPT.

The median age was 28.0 years (IQR, 25.0, 31.0) and the median prepregnancy body mass index was 28.7 kg/m^2^ (IQR, 24.1, 33.7). The median duration of T1D was 17.0 years (IQR, 11.0, 22.0). Most women self‐identified as non‐Hispanic White race and ethnicity (94.8%), and 24.8% had Medicaid health insurance. Women using HCL versus SAPT were more likely to have an Ominipod 5® (41.0% vs. 30.6%) and Tandem t:slim X2® (42.9% vs. 34.7%) than to have a Medtronic MiniMed 780G® (16.2% vs. 34.7%) (*p* = 0.03). Demographic characteristics did not vary by HCL versus SAPT (Table 1).

**TABLE 1:** Pregnant women with type 1 diabetes using HCL versus SAPT (*N* = 154).

		SAPT *N* = 49	HCL *N* = 105	
	Overall	*N* (%)	*N* (%)	*p* value^a^
Age, years, median (IQR)	28.0 (25.0, 31.0)	27.0 (24.0, 32.0)	29.0 (26.0, 31.0)	0.4
Type of insulin pump				**0.03**
Omnipod 5	58 (37.7)	15 (30.6)	43 (41.0)	
Tandem T‐Slim X2	62 (40.3)	17 (34.7)	45 (42.9)	
Medtronic 780	34 (22.1)	17 (34.7)	17 (16.2)	
Age at diabetes diagnosis, years, median (IQR)	11.0 (7.0, 17.0)	11.0 (8.0, 18.0)	11.0 (7.0, 16.0)	0.3
Gestational age at initiation of prenatal care, weeks, median (IQR), *N* = 152	8.0 (7.3, 9.3)	8.0 (7.5, 9.3)	8.0 (7.1, 9.3)	>0.9
Diabetes duration, years, median (IQR)	17.0 (11.0, 22.0)	14.0 (9.0, 22.0)	18.0 (13.0, 22.0)	0.1
Race/ethnicity, self‐reported, non‐Hispanic White	146 (94.8)	46 (93.9)	100 (95.2)	0.7
Medicaid health insurance, *N* = 153	38 (24.8)	14 (28.6)	24 (23.1)	0.8
Prepregnancy body mass index, kg/m^2^, median (IQR)	28.7 (24.1, 33.7)	28.6 (24.1, 32.8)	29.0 (24.7, 33.8)	0.8
Modified white diabetes classification				0.2
B (onset ≥ 20 years and duration < 10 years)	26 (16.9)	13 (26.5)	13 (12.4)	
C (onset 10‐19 years or duration 10‐19 years)	53 (34.4)	12 (24.5)	41 (39.0)	
D (onset < 10 years, duration > 20 years, retinopathy/HTN)	65 (42.2)	21 (42.9)	44 (41.9)	
F (nephropathy)	2 (1.3)	1 (2.0)	1 (1.0)	
R (proliferative retinopathy)	7 (4.5)	2 (4.1)	5 (4.8)	
RF (nephropathy and proliferative retinopathy)	1 (0.6)	0 (0.0)	1 (1.0)	
Chronic hypertension, *N* = 153	27 (17.6)	9 (18.4)	18 (17.3)	0.9
Baseline creatinine, median (IQR), mg/dL, *N* = 150	0.6 (0.5, 0.7)	0.6 (0.5, 0.7)	0.6 (0.5, 0.7)	0.4
Early pregnancy A1c, *N* = 153				
Median A1c% (IQR)	6.7 (6.2, 7.5)	7.0 (6.2, 8.5)	6.6 (6.2, 7.2)	0.06
A1c < 6.0%	30 (19.6)	10 (20.8)	20 (19.0)	0.8
A1c < 6.5%	56 (36.6)	13 (27.1)	43 (41.0)	0.1
CGM metrics,^b^ early pregnancy, median (IQR)				
Mean glucose, *N* = 147 (mg/dL)	148.0 (131.5, 169.5)	145.0 (132.0, 169.0)	150.0 (131.8, 170.0)	0.6
Time in range (IQR), *N* = 129 (%)	64.0 (49.0, 76.0)	64.0 (49.0, 76.0)	66.0 (49.5, 76.0)	0.7
Time above range (IQR), *N* = 129 (%)	31.0 (19.0, 49.0)	31.0 (15.0, 47.0)	30.0 (20.0, 49.5)	0.4
Time below range (IQR), *N* = 129 (%)	2.0 (1.0, 5.0)	4.0 (1.0, 9.0)	2.0 (1.0, 4.0)	**0.02**
Standard deviation, *N* = 139	50.0 (40.5, 63.0)	53.0 (35.5, 63.5)	50.0 (41.8, 62.2)	0.6
Adjunctive medication strategies during pregnancy				
Basal insulin	18 (11.7)	2 (4.1)	16 (15.2)	
Metformin	12 (7.8)	2 (4.1)	10 (9.5)	**0.04**

Abbreviations: CGM, continuous glucose monitoring; HCL, hybrid closed‐loop; HTN, hypertension; IQR, interquartile range; SAPT, sensor‐augmented pump therapy.

^a^ Bolded results are statistically significant using a higher threshold given sample size (*p* < 0.10).

^b^ Time in range: 65–140 mg/dL, time above range: >140 mg/dL, time below range: <65 mg/dL.

During the week before initiation of prenatal care, the median pregnancy‐specific TIR was 64.0% (IQR, 49.0, 76.0) and the median A1c was 6.7% (IQR, 6.2, 7.5). Glycemic characteristics did not vary by HCL versus SAPT (Table 1) with the exception of TAR, which was lower in those intending to use HCL than SAPT (*p* = 0.02). Women using HCL were more likely to require the addition of basal insulin and metformin compared with those using SAPT (*p* = 0.04).

Of the 105 women who intended to use HCL, 33 (31.4%) later changed to SAPT at a median gestational age of 28.0 weeks (IQR, 22.3, 31.0); of the 49 women who intended to use SAPT, 7 (14.3%) later changed to HCL at a median gestational age of 26.0 weeks (IQR, 23.5, 27.0). The median duration of HCL use from initiation of prenatal care to delivery for women who only used HCL was 29.1 weeks (IQR, 27.3, 30.2), who switched from HCL to SAPT was 8.6 weeks (IQR, 5.4, 15.6), and who switched from SAPT to HCL was 9.9 weeks (IQR, 9.3, 13.2). No women exclusively switched to multiple daily injections of insulin.

In adjusted analyses, women using HCL had neonates with a higher birthweight (3640 vs. 3430 g; adj. beta, 271.98; 95% CI, 0.31, 543.65) and mean sensor glucose the week prior to delivery (150.0 vs. 145.0 mg/dL; adj. beta, 13.36; 95% CI, 0.79, 25.93) and lower TBR compared with those using SAPT (2.0 vs. 4.0%; adj. beta, −3.73; 95% CI, −6.52, −0.93) (Table 2). Other pregnancy and glycemic outcomes were similar between groups.

**TABLE 2:** Association between HCL versus SAPT and adverse pregnancy and glycemic outcomes (*N* = 154).

	SAPT (Reference) *N* (%) *N* = 49	HCL *N* (%) *N* = 105	Unadjusted analysis (95 CI%)	Adjusted analysis (95 CI%)^a^, ^b^
Pregnancy outcomes^a^				
Cesarean delivery	32 (65.3)	68 (64.8)	0.99 (0.85, 1.16)	0.98 (0.74, 1.30)
Primary	22 (68.8)	35 (51.5)	‐	‐
Repeat	10 (31.2)	33 (48.5)	‐	‐
Hypertensive disorders of pregnancy, *N* = 150	32 (65.3)	60 (59.4)	0.91 (0.76, 1.09)	0.92 (0.69, 1.22)
Preterm birth < 37 weeks, *N* = 152	22 (44.9)	29 (28.2)	0.63 (0.24, 1.63)	0.65 (0.40, 1.07)
Spontaneous	6 (27.3)	4 (13.8)	‐	‐
Iatrogenic	16 (72.7)	25 (86.2)	‐	‐
Large‐for‐gestational‐age at birth, *N* = 147	20 (41.7)	49 (49.5)	1.19 (0.68, 2.06)	1.30 (0.84, 2.03)
Birthweight, grams, median (IQR), *N* = 147	3,430.0 (2,955.0, 3,792.0)	3,640.0 (3,240.0, 3,990.0)	257.00 (−32.14, 546.13)	**271.98 (0.31, 543.65)**
>4500 g	3 (6.2)	12 (12.1)	‐	‐
>4000 g	8 (16.7)	23 (23.2)	‐	‐
NICU admission, *N* = 152	22 (45.8)	38 (36.5)	0.80 (0.45, 1.41)	0.83 (0.54, 1.28)
Neonatal hypoglycemia, *N* = 151	36 (75.0)	64 (62.1)	**0.83 (0.74, 0.93)**	0.86 (0.67, 1.11)
Neonatal hyperbilirubinemia, *N* = 151	13 (27.1)	20 (19.4)	0.72 (0.08, 6.32)	0.73 (0.38, 1.41)
Respiratory distress syndrome, *N* = 151	17 (35.4)	29 (28.2)	0.79 (0.26, 2.39)	0.81 (0.47, 1.40)
Glycemic outcomes^a^				
Last A1c ≤ 6.0% before delivery, *N* = 153	21 (42.9)	41 (39.4)	0.92 (0.48, 1.78)	0.76 (0.54, 1.08)
CGM metrics before delivery^c^				
Mean glucose (mg/dL), *N* = 147	145.0 (132.0, 169.0)	150.0 (131.8, 170.0)	5.62 (−7.28, 18.52)	**13.36 (0.79, 25.93)**
Time in range (%), *N* = 129	64.0 (49.0, 76.0)	66.0 (49.5, 76.0)	1.98 (−5.71, 9.67)	2.06 (−4.05, 8.18)
Time above range (%), *N* = 129	31.0 (15.0, 47.0)	30.0 (20.0, 49.5)	3.02 (−5.11, 11.15)	2.96 (−3.43, 9.35)
Time below range (%), *N* = 129	4.0 (1.0, 9.0)	2.0 (1.0, 4.0)	**−3.75 (−6.56, −0.95)**	**−3.73 (−6.52, −0.93)**
Standard deviation, *N* = 139	53.0 (35.5, 63.5)	50.0 (41.8, 62.2)	1.59 (−6.31, 9.49)	5.78 (−0.67, 12.23)

*Note*: Bolded results are statistically significant.

Abbreviations: CI, confidence interval; HCL, hybrid closed‐loop; IQR, interquartile range; SAPT, sensor‐augmented pump therapy.

^a^ An Inverse‐Probability‐of‐Treatment Weighting (IPTW) analytic approach was used to address confounding by HCL manufacturer/type, duration of T1D, and early pregnancy A1c, and clustered for interpersonal correlation for women with data from multiple pregnancies.

^b^ Categorical outcomes (relative risk, 95% CI), and continuous outcomes (Beta coefficient, 95% CI).

^c^ Time in range: 63–140 mg/dL, time above range: >140 mg/dL, time below range: <63 mg/dL.

The above results were similar when the exposure was secondarily assessed as any HCL use in pregnancy versus SAPT (Appendix Table 1 in the Supporting Information).

## 4 CONCLUSION

Among a US cohort with T1D enrolled in a prenatal and diabetes care program, women intending to use off‐label HCL had slightly higher birthweight and mean glucose and lower TBR before delivery compared with those using SAPT. Most pregnancy and glycemic outcomes used in clinical practice did not differ between groups. While the above continuous measures were statistically significant, these differences may not necessarily be clinically significant. Whether a slightly higher birthweight with non‐pregnancy‐specific HCL reflects inferior glycemic management and affects clinical outcomes requires further study.

This real‐world contemporary US cohort complements recent data from two international clinical trials and may help to inform person‐centered shared decision‐making. In the AiDAPT trial, use of the CamAPS FX system with a pregnancy‐specific algorithm was associated with improved glycemic control [4]. The CRISTAL RCT found that MiniMed 780G with a non‐pregnancy algorithm resulted in more frequent overnight TIR, lower TBR, and improved treatment satisfaction [2]. The rate of TIR in both trials (66%–68%) was similar to the current study. Of note, both trials were not powered for pregnancy outcomes, but the AiDAPT trial found less excessive gestational weight gain and neonatal hypoglycemia with HCL [4]. A recent small US observational study found that women who used HCL with a pregnancy‐specific predictive algorithm had more frequent late pregnancy TIR than those who used SAPT [8]. The current analysis did not examine CGM data across the entire pregnancy, and further analyses assessing longitudinal changes in CGM metrics with functional data analysis may be able to better capture changing glucose trajectories across pregnancy in response to changes in patient engagement with diabetes technology [9].

Strengths of this study were its sample size larger than the two most recent clinical trials and contemporary data from a clinical setting. The current study includes outcomes for women using both the Tandem T‐Slim X2 and Omnipod 5 HCL systems, the two pumps most frequently used in the United States.

Study limitations include lack of data on a broader set of behavioral factors, social determinants of health, and measures of healthcare utilization that may impact glycemic management and pregnancy outcomes. Glycemic data including CGM measures were not assessed across pregnancy but at discrete clinically relevant time points. Data on insulin pump use prior to the current pregnancy were not collected; however, the current analysis used an analytic approach to account for confounding by indication. Although the current study was a single‐center retrospective analysis, these data can inform clinical practice and the increasing off‐label use of HCL in pregnancy.

Given the persistently high rates of adverse pregnancy outcomes for women with T1D, HCL use may benefit maternal and child health if it allowed for improvement in glycemic management and pregnancy outcomes. Evaluating how HCL and pregnancy‐specific algorithms can be used to promote maternal and child health could be most helpful for pregnant women with T1D and should be a research priority.

## CONFLICT OF INTEREST STATEMENT

The authors declare no conflicts of interest.

## Supporting information



Supporting Information

## Data Availability

The data that support the findings of this study are available on request from the corresponding author. The data are not publicly available due to privacy or ethical restrictions.
