# Supplementary material for: Pregnancy and glycemic outcomes with hybrid closed‐loop insulin delivery versus sensor‐augmented pump therapy among women with type 1 diabetes
Source: Pregnancy (Hoboken). 2026 Aug 31;2(5):e70392. doi: 10.1002/pmf2.70392 (PMC13527856; doi:10.1002/pmf2.70392)
Supplement: Supplementary file 1 [file PMF2-2-e70392-s001.docx]

| **Appendix Table 1. Sensitivity analysis association between any HCL in pregnancy versus SAPT only and adverse pregnancy and glycemic outcomes (N=154)** | | | | |
| --- | --- | --- | --- | --- |
|  | **SAPT (Reference)**  **N (%)** | **Any HCL**  **N (%)** | **Unadjusted analysis**  **(95 CI%)** | **Adjusted analysis**  **(95 CI%)^1,2^** |
|  | **N=42** | **N=112** |  |  |
| **Maternal Outcomes** |  |  |  |  |
| Cesarean delivery | 26 (70.3) | 72 (64.3) | 0.91 (0.81, 1.04) | 0.89 (0.69, 1.16) |
| Hypertensive disorders of pregnancy, N=145 | 26 (70.3) | 65 (60.2) | 0.86 (0.74, 1.00) | 0.94 (0.66, 1.33) |
| Preterm birth <37 weeks, N=147 | 19 (51.4) | 31 (28.2) | 0.55 (0.22, 1.37) | 0.59 (0.35, 1.00) |
| Large for gestational age, N=142 | 15 (41.7) | 53 (50.0) | 1.20 (0.69, 2.10) | 1.29 (0.79, 2.13) |
| Birthweight (grams), IQR, N=142 | 3,340.5 (2,937.5, 3,805.0) | 3,640.0 (3,242.5, 3,990.0) | 315.46 (-15.78, 646.71) | **333.11 (59.86, 606.35)** |
| NICU Admission, N=147 | 18 (48.6) | 42 (38.2) | 0.78 (0.47, 1.32) | 0.78 (0.49, 1.23) |
| Neonatal hypoglycemia, N=146 | 26 (70.3) | 70 (64.2) | 0.91 (0.81, 1.03) | 1.06 (0.76, 1.48) |
| Neonatal hyperbilirubinemia, N=146 | 10 (27.0) | 22 (20.2) | 0.75 (0.08, 6.71) | 0.73 (0.37, 1.43) |
| Respiratory distress syndrome, N=146 | 14 (37.8) | 32 (29.4) | 0.78 (0.29, 2.11) | 0.79 (0.53, 1.18) |
| **Glycemic outcomes** |  |  |  |  |
| Last A1c ≤6.0% before delivery, N=148 | 14 (37.8) | 44 (39.6) | 1.05 (0.44, 2.49) | 0.79 (0.53, 1.18) |
| CGM Metrics^2^, last before delivery |  |  |  |  |
| Mean glucose (mg/dL), N=142 | 147.0 (133.5, 171.0) | 150.0 (132.0, 170.0) | 1.38 (-11.50, 14.26) | **12.02 (0.11, 23.92)** |
| Time in range (%) (IQR), N=124 | 60.0 (49.0, 76.0) | 63.0 (48.5, 75.5) | 3.77 (-4.38, 11.91) | 3.16 (-3.36, 9.69) |
| Time above range (%) (IQR), N=124 | 31.0 (15.0, 47.0) | 31.0 (20.0, 49.5) | 2.27 (-6.36 , 10.89) | 2.90 (-3.44, 9.25) |
| Time below range (%) (IQR), N=124 | 4.0 (1.0, 12.0) | 2.0 (1.0, 4.0) | **-4.73 (-8.30, -1.15)** | **-4.71 (-8.25, -1.17)** |
| Standard deviation, N=134 | 58.0 (37.5, 64.5) | 50.0 (42.0, 62.5) | -0.86 (-9.33, 7.62) | 4.63 (-2.10, 11.37) |

^1^An Inverse-Probability-of-Treatment Weighting (IPTW) analytic approach was used to address confounding by HCL manufacturer/type, duration of T1D, and early pregnancy A1c, and clustered for interpersonal correlation for women with data from multiple pregnancies.

^2^Categorical outcomes (Relative Risk, 95% CI), and continuous outcomes (Beta coefficient, 95% CI)

^3^Time in range: 65 to 140 mg/dL, time above range: >140 mg/dL, time below range: <65 mg/dL

Bolded results are statistically significant.
